# Supplementary material for: Cell-type-specific co-expression inference from single cell RNA-sequencing data
Source: Nat Commun. 2023 Aug 10;14:4846. doi: 10.1038/s41467-023-40503-7 (PMC10415381; doi:10.1038/s41467-023-40503-7)
Supplement: Supplementary file 1 — Supplementary information [file 41467_2023_40503_MOESM1_ESM.pdf]

Supplementary Information for "Cell-type-specific co-expression inference from single cell RNA-sequencing data"

Chang Su, Zichun Xu, Xinning Shan, Biao Cai, Hongyu Zhao, Jingfei Zhang

Contents

Supplementary Methods **2**

Details of CS-CORE Algorithm . . . 2

Details of simulating expression data from model (1) . . . 2

Details of extracting co-expressed gene modules . . . 3

Details of enrichment and reproducibility analyses . . . 4

GO and Reactome Enrichment analysis . . . 4

Overlap with known TF-target gene pairs . . . 4

Reproducibility analysis . . . 4

Evaluation of empirical p-values . . . 5

Supplementary Notes **5**

Supplementary Discussion **6**

Supplementary Tables **8**

Supplementary Figures **9**

Supplementary Methods

Details of CS-CORE Algorithm

In Algorithm 1, $f_{\mu_j}(w_{ij}), f_{\sigma_{jj}}(\mu_j, h_{ij}), f_{\sigma_{jj'}}(\mu_j, \mu_{j'}, g_{ijj'})$ are the weighted least squares operators for estimating μ_j, σ_{jj} and $\sigma_{jj'}$, respectively. They are defined as follows:

$$
\begin{aligned}
f_{\mu_j}(w_{ij}) &= \sum_i (s_i x_{ij}) w_{ij} / \sum_i s_i^2 w_{ij}, \\
f_{\sigma_{jj}}(\mu_j, h_{ij}) &= \sum_i s_i^2 [(x_{ij} - s_i\mu_j)^2 - s_i\mu_j] h_{ij} / \sum_i s_i^4 h_{ij}, \\
f_{\sigma_{jj'}}(\mu_j, \mu_{j'}, g_{ijj'}) &= \sum_i s_i^2 (x_{ij} - s_i\mu_j)(x_{ij'} - s_i\mu_{j'}) g_{ijj'} / \sum_i s_i^4 g_{ijj'}.
\end{aligned}
$$

Details of simulating expression data from model (1)

In (1), we considered the following expression-measurement model:

$$(z_{i1}, \dots, z_{ip}) \sim F_p(\boldsymbol{\mu}, \boldsymbol{\Sigma}), \quad x_{ij}|z_{ij} \sim \text{Poisson}(s_i z_{ij}),$$

where $F_p(\boldsymbol{\mu}, \boldsymbol{\Sigma})$ is a nonnegative p-variate distribution with mean vector $\boldsymbol{\mu} = (\mu_1, \dots, \mu_p)$ and covariance matrix $\boldsymbol{\Sigma} = (\sigma_{jj'})_{p\times p}$. For the experiment in Figure 2, we set $\boldsymbol{\Sigma}$ to be an identity matrix and simulated gene expressions independently. For experiments in Figures 3B-C and 4A, we used the copula method described in the Methods section to simulate from the above model. Next, we discuss how the parameters were set in these experiments.

To select values of μ_j, σ_{jj} and s_i that resemble real data, we utilized the single cell data on excitatory neurons from Lau et al. [1]. Specifically, for sequencing depths s_i, we sampled from the observed values in real data. For mean and variance parameters μ_j and σ_{jj}, we fitted a negative binomial generalized linear model [2] that estimated μ_j and $\text{CV}_j^2 = \sigma_{jj}/\mu_j^2$ across genes using maximum likelihood. We fitted $\log_{10}(\text{CV}_j^2)$ as a function of $\log_{10}(\mu_j)$ with kernel smoothing regression [3], denoted as $\hat{f}_j(\cdot)$, to reduce variability in CV_j's [4, 5, 6, 7]. Finally, we used estimates of μ_j and $\sigma_{jj} = 10^{\hat{f}_j(\mu_j)} \times \mu_j^2$ as the parameters in the marginal Gamma distributions.

In Figure 2, we generated $n = 1{,}000$ cells and focused on a gene pair that ranked 269 and 351 among 28,412 genes in excitatory neurons from [1].

In Figures 3B-C, we generated $n = 5,000$ cells on $p = 500$ genes. We set $\mu_j, \sigma_{jj}, j = 1, \dots, 500$ in simulation to be the estimates for the top 500 highly expressed genes. To specify a matrix R that resembles real data in Figure 3B, we first applied CS-CORE to estimate the co-expression matrix for the top 500 highly expressed genes in excitatory neurons from [1] and only focused on significantly co-expressed gene pairs (BH-adjusted p-values < 0.05).

We further set co-expression estimates with absolute values less than 0.5 to 0 to encourage sparsity. Finally, we scaled off-diagonal entries by 3.1 such that the scaled matrix was positive definite.

In Figure 4A, we generated $n = 2,000$ cells on $p = 100$ genes. We selected 100 genes randomly from the top 2000 highly expressed genes and set $\mu_j, \sigma_{jj}, j = 1, \ldots, 100$ in the simulation to the estimated μ_j's and σ_{jj}'s from these genes. To generate a co-expression matrix R with the clustering structure in Figure 4A, we adopted the degree-corrected Stochastic Block model [8] and used `BlockModel.Gen` in R package *randnet* (v.0.1) to simulate a binary network of size 100, where we specified four co-expressed modules and set the ratio of within-block edges and between-block edges to be 1,000. We further set the node degree (i.e. sum of co-expressed genes) distribution to be a power-law distribution with power equal to 4 and mean equal to 20. Given the binary network, we then simulated co-expression values from a truncated standard normal distribution between 0.9 and 1. Finally, we used the software from [9] to obtain a closest positive definite correlation matrix and removed five genes that had minimal connections with other genes in their assigned clusters. We note that the mean expression levels of genes were randomly sampled from the top 2,000 highly expressed genes in real data. Correspondingly, the cluster structure was only present in the correlation matrix and gene expression levels were similar across gene clusters, with mean expression levels (on the scale of $\log_{10} \mu_j + 3$) -0.55 with an sd=0.36 for cluster 1, -0.55 with an sd=0.45 for cluster 2, -0.57 with an sd=0.39 for cluster 3 and -0.70 with an sd=0.26 for cluster 4.

The software for Normalisr, Noise regularization, propr, sctransform and SpQN require the entire gene count matrix as input, for example, to calculate sequencing depth per cell. In this case, we further simulated independent counts with marginal statistics resembling real data for genes that were not included in Figures 2, 3B-C and 4A.

Details of extracting co-expressed gene modules

To obtain co-expressed gene modules, we estimated co-expressions, obtained significant co-expressed gene pairs by extracting the pairs with BH-adjusted p-values < 0.05, and performed clustering analyses following WGCNA [10] with the soft-thresholding power set to 1. We used the analytic p-values to select significant pairs for CS-CORE and ρ-sctransform and the empirical p-values to select significant pairs for ρ-analytic PR as it has inflated type-I error (Figure 1, Supplementary Methods "Evaluation of empirical p-values").

Details of enrichment and reproducibility analyses

GO and Reactome Enrichment analysis

Given a gene set identified via clustering analysis, for enrichment in biological pathways, we performed enrichment analysis of Gene Ontology (GO) using R package clusterProfiler (v.4.2.2) from [11] and Reactome Pathway Database (Reactome) using R package ReactomePA (v.1.38.0) from [12] with background genes set to all genes that were used in the clustering analysis. We reported significantly enriched GO terms which had BH-adjusted p-values less than 0.05.

Overlap with known TF-target gene pairs

Given an scRNA-seq data set and a co-expression estimation method, for assessing whether the method can identify known TF-target gene pairs, we took the union of the top 5,000 highly expressed genes and genes in the TRRUST database [13], calculated the p-values in testing for co-expressed gene pairs using CS-CORE, ρ-analytic PR or ρ-sctransform and counted the number of significant gene pairs that overlapped with the TRRUST database across different p-value cutoffs.

The analytical p-values were evaluated for CS-CORE and ρ-sctransform as these two methods have appropriate type-I error control, while the empirical p-values were evaluated for ρ-analytic PR as it suffers from inflated type-I errors (Supplementary Methods "Evaluation of empirical p-values"). We only considered p-value cutoffs 10^{-2} and 10^{-3} for ρ-analytic PR due to the computational demand for evaluating empirical p-values.

Reproducibility analysis

To evaluate the reproducibility of gene pairs identified with CS-CORE, ρ-analytic PR and ρ-sctransform in five brain cell types (Figure 5A and Supplementary Fig. 8, 9A-B), we counted the number of reproduced pairs across different p-value cutoffs between two sets of independent snRNA-seq data sets on the brain. Specifically, for each cell type, we used the cells on control subjects from [1] and [14], took the intersection of top 5,000 highly expressed genes between these two data sets, computed co-expression p-values for the intersected genes and compared significant gene pairs inferred in these two data sets (Figure 5A, Supplementary Fig. 9A). We also used the cells on control subjects from [1] and [15] and compared significant gene pairs similarly (Supplementary Fig. 8, 9B). To evaluate the reproducibility in five immune cell types in PBMC (Supplementary Fig.11-12), we used cells of control subjects from two independent PBMC data sets [16] and [17] and counted the number of reproduced

gene pairs in each cell type following a similar procedure. The evaluation of p-values and the choice of p-value cutoffs are similar to that in Supplementary Methods "Overlap with known TF-target gene pairs".

Evaluation of empirical p-values

We evaluated empirical p-values for methods that do not offer statistical test (propr, SpQN) and for methods that offer tests but suffer from inflated type I errors (Noise regularization, Pearson correlations of log normalized data, Spearman correlations of log normalized data and ρ-analytic PR).

The detailed procedure of evaluating the empirical p-value is as follows: For each count matrix, 100 sets of independent UMI counts that resemble the marginal statistics of the counts were simulated (Methods and Supplementary Methods). The empirical null distribution was constructed by the co-expression estimates on these simulated null data. The empirical p-value was then evaluated by counting the proportion of 100 sets of null estimates that were larger in magnitude than the estimates on observed data. The evaluation was repeated for 100 data replicates in Supplementary Fig. 2 and for 1,000 data replicates in Supplementary Fig. 9,12. We did not evaluate empirical p-values for locCSN and baredSC due to their extreme computational demands. The average empirical power was used to assess the statistical power of a method.

Supplementary Notes

Bias in baredSC estimates. On null data with independent gene expressions, baredSC yielded positive estimates that significantly deviated from zero in both permuted data (Figure 1) and simulated data (Supplementary Fig. 1), even when the sequencing depths were set to be constant across cells. This suggested that the estimation bias in baredSC was due to reasons other than sequencing depth confounding. On simulated data with correlated gene expressions, baredSC was found to have an underestimation bias (Figure 3A). We speculate two possible causes for these biases from baredSC in our experiments. First, the assumption of Gaussian mixtures used to fit log transformed underlying expression levels in baredSC may lead to biased estimates when the underlying distribution cannot be well approximated by a Gaussian mixture model. Second, in baredSC, the MCMC sampling used in inference and the numerical integration used to approximate the likelihood function are both subject to approximation errors.

GO enrichment results of SpQN. While SpQN identified modules on (trans-)synaptic signalling in neurons and oligodendrocytes that were not found enriched among CS-CORE modules (Supplementary Data 1), we note that SpQN modules were generally much larger in sizes compared to CS-CORE and the other two methods and larger gene modules tended to yield more significant GO terms. For example, across five major brain cell types in the dataset from [1], the modules identified from SpQN estimates have a median of 363 genes, while the modules from CS-CORE, ρ-sctransform and ρ-analytic PR have a median of 70, 71 and 79.5 genes, respectively.

Weight selection in IRLS. Under our modeling framework, optimal weights, in terms of minimizing variances, for the variance and covariance weighted least squares estimators are difficult to derive analytically as they require fourth moment quantities such as $\mathrm{Var}(x_{ij}^2)$ or $\mathrm{Var}(x_{ij}x_{ij'})$, where x_{ij} is the UMI count from gene j in sample i. These higher moments need to be derived under explicit parametric assumptions on the underlying expression levels. In CS-CORE, we do not place such assumptions as they can limit the flexibility of our approach. Instead, we set weights for the variance and covariance estimators to be $\{\mathrm{Var}(x_{ij})\}^{-2}$ and $\{\mathrm{Var}(x_{ij})\mathrm{Var}(x_{ij'})\}^{-1}$ based on the rationale that, when estimating σ_{jj} or $\sigma_{jj'}$, the sample with a larger variance in counts should have a lower weight.

Moreover, these selected weights were found to perform similarly as the weights derived under explicit parametric assumptions. For example, under a negative binomial assumption on x_{ij}'s, it can be shown that the optimal weight of the variance estimator is $\{\mathrm{Var}(x_{ij}) + (2 + 6\sigma_{jj}/\mu_j^2) \cdot \mathrm{Var}(x_{ij})^2\}^{-1}$. This is similar to our selected weight of $\{\mathrm{Var}(x_{ij})\}^{-2}$ if σ_{jj}/μ_j^2's are similar across genes. In our experiments, we found the derived weights under the negative binomial assumption were slower to compute due to their involved formulas and yielded very similar numerical performances.

Supplementary Discussion

We note that CS-CORE should be applied to cells from the same cell type, while the inferred co-expressions may be spurious when cells from different cell types are considered. In Equation (1), it is assumed that the underlying expression levels $(z_{i1}, \ldots, z_{ip})$'s are identically distributed following $F_p(\boldsymbol{\mu}, \boldsymbol{\Sigma})$ for all cells $i = 1, \ldots, n$ considered. This assumption is appropriate for modelling cells from the same cell type, in which case $\boldsymbol{\Sigma}$ is the cell-type-specific co-expression that characterizes the biological functions and pathways in a specific cell type. In contrast, spurious co-expressions may arise when cells from different cell types are considered, due to the violation of the identically distributed assumption. For example,

if two marker genes for cell type 1 are independently expressed and cells from both cell type 1 and other cell types are considered, this gene pair may appear to be spuriously co-expressed as they are both expressed in cell type 1 and not expressed in other cell types. In this case, the co-expressions are confounded by the changes in mean expression levels. In general, when applied to cells from different cell types, the inferred co-expressions is challenging to interpret and can be misleading for investigating cell-type-specific biological functions.

Supplementary Tables

For the differentially co-expressed gene module presented in Figure 6, we further used hierarchical clustering to generate three sub-modules and annotated the functions of each sub-module with GO and Reactome enrichment analyses (Supplementary Methods), with background set to all genes in the databases to interpret the gene sets. Representative GO terms and Reactome pathways are reported for each sub-module.

Supplementary Table 1: Gene Ontology and Reactome enrichment analysis for the three sub-modules in Figure 6.

Module	Pathway	ID	Description	Adjusted p-value	Gene ratio
1. Toll-like receptor signalling	GO	GO:0050665	hydrogen peroxide biosynthetic process	2.13e-03	2/5
		GO:1903428	positive regulation of reactive oxygen species biosynthetic process	2.13e-03	2/5
		**GO:0034121**	**regulation of toll-like receptor signaling pathway**	**5.55e-03**	**2/5**
	Reactome	R-HSA-3299685	Detoxification of Reactive Oxygen Species	1.14e-02	2/6
2. Interferon signalling	GO	GO:0009615	response to virus	7.22e-23	17/21
		GO:0051607	defense response to virus	3.14e-21	15/21
		GO:0140546	defense response to symbiont	3.14e-21	15/21
	Reactome	R-HSA-909733	Interferon alpha/beta signaling	2.03e-14	9/18
		**R-HSA-913531**	**Interferon Signaling**	**3.27e-14**	**11/18**
		R-HSA-1169410	Antiviral mechanism by IFN-stimulated genes	2.96e-08	6/18
3. Antigen Presentation	GO	**GO:0019885**	**antigen processing and presentation of endogenous peptide antigen via MHC class I**	**4.42e-04**	**3/18**
		GO:0002483	antigen processing and presentation of endogenous peptide antigen	4.42e-04	3/18
		GO:0019883	antigen processing and presentation of endogenous antigen	7.87e-04	3/18
	Reactome	R-HSA-1236977	Endosomal/Vacuolar pathway	3.29e-04	3/19
		R-HSA-983170	Antigen Presentation: Folding, assembly and peptide loading of class I MHC	2.26e-03	3/19
		**R-HSA-9694516**	**SARS-CoV-2 Infection**	**1.59e-02**	**3/19**

Supplementary Figures

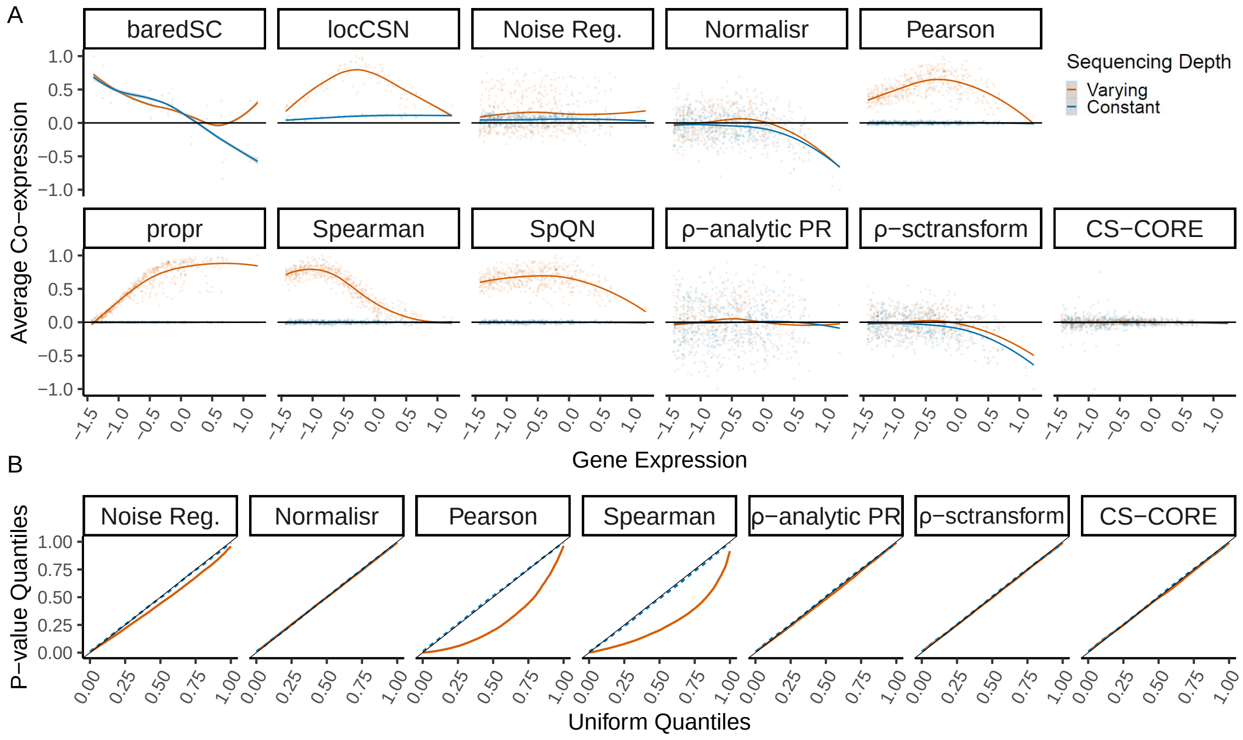

Supplementary Figure 1: Validation of CS-CORE using simulated scRNA-seq data with independent gene expressions. We selected genes and cells as in Figure 1 and simulated data as described in Methods with a diagonal correlation matrix and marginal negative binomial distributions. Results from simulated data with varying and constant sequencing depths are colored with light red and blue, respectively. **A** Scatter plots with fitted curves showing mean expression levels (x-axis) and average co-expression (y-axis) of each gene with co-expression estimated using baredSC, locCSN, Noise Regularization, Normalisr, Pearson correlation of log normalized data (Pearson), propr, Spearman correlation of log normalized data (Pearson), SpQN, ρ-analytic PR, ρ-sctransform, and CS-CORE. Average co-expressions are re-scaled by the maximum value to aid comparison. The mean expression levels are plotted at the scale of $\log_{10}\mu_j+3$ for μ_j defined in Equation (1). **B** Q-Q plots comparing p-values for testing co-expressions of gene pairs against Uniform(0,1) using seven methods with statistical tests, including Noise Regularization, Normalisr, Pearson correlation of log normalized data (Pearson), Spearman correlation of log normalized data (Pearson), ρ-analytic PR, ρ-sctransform and CS-CORE. Source data are provided as a Source Data file.

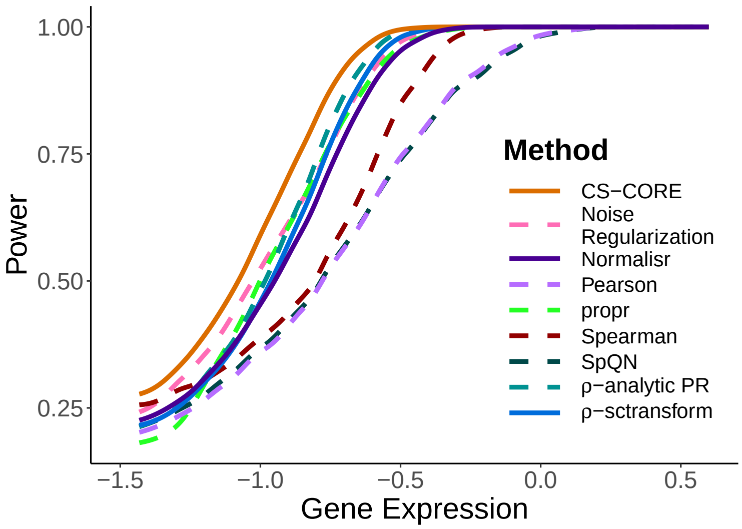

Supplementary Figure 2: Statistical power in detecting co-expression at different gene expression levels. For the three methods with appropriate type-I error controls in Figure 1B (CS-CORE, Normalisr and ρ-sctransform), the analytical power was evaluated based on the proposed statistical tests and plotted with solid lines. For methods that offer statistical tests but suffer from inflated type-I errors (Noise regularization, Pearson correlations of log normalized data, Spearman correlations of log normalized data and ρ-analytic PR) or methods that do not offer statistical tests (propr, SpQN), the empirical power was evaluated based on empirical p-values as described in Supplementary Methods and plotted with dashed lines. Gene expressions were simulated under the same setting as in Figure 3A. The lines show the average power across expression levels fitted by kernel smoothing. Source data are provided as a Source Data file.

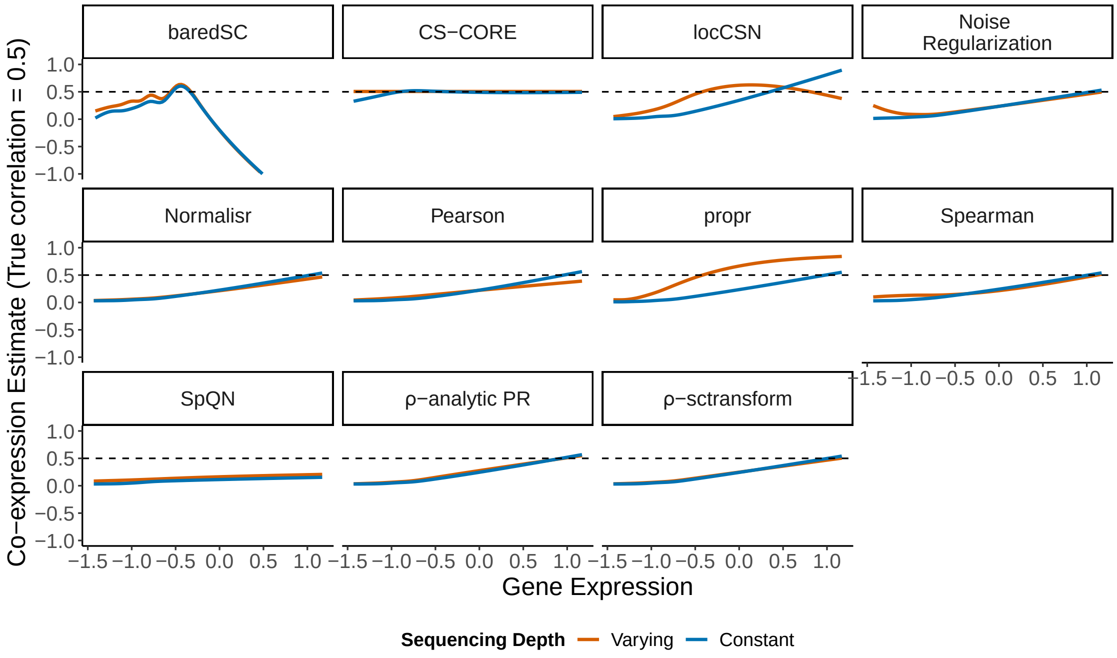

Supplementary Figure 3: Validation of CS-CORE co-expression estimates using simulated data with constant and varying sequencing depths, compared to baredSC, locSCN, Noise Regularization, Normalisr, Pearson correlation of log normalized data, propr, Spearman correlation of log normalized data, SpQN, ρ-analytic PR and ρ-sctransform under a similar setting as Figure 3A. Curve-fitted co-expression estimates are plotted against geometric mean expression levels on gene pairs simulated with a true correlation of 0.5 (5,000 genes and 1,000 cells). Data were simulated as described in Methods. Source data are provided as a Source Data file.

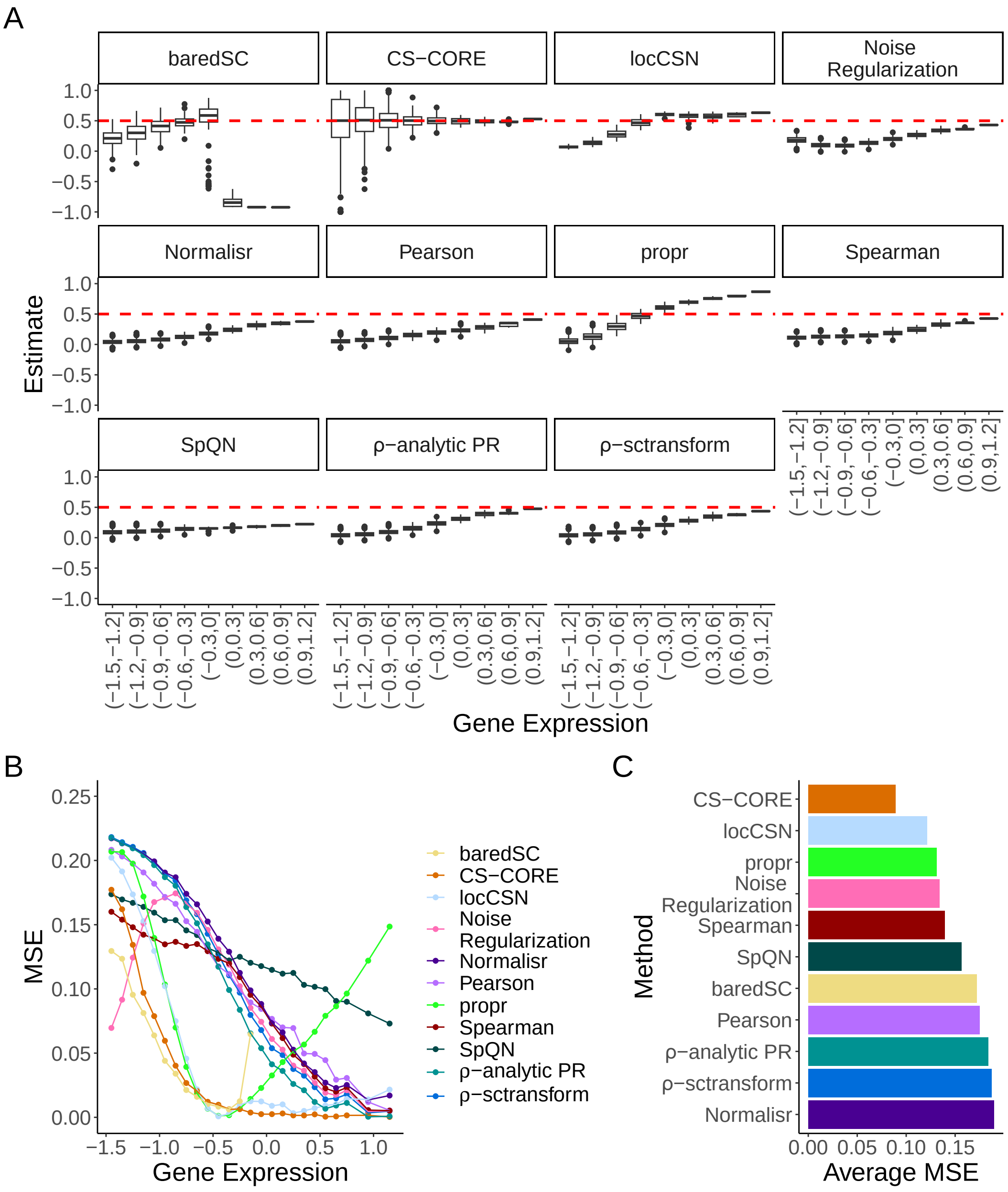

Supplementary Figure 4: (Caption on the next page.)

Supplementary Figure 4: (Figure on the previous page.) Evaluation of variance and mean squared errors (MSE) of co-expression estimator on gene pairs simulated with a true correlation of 0.5 (5,000 genes and 1,000 cells) under the same setting as Figure 3A. **A** Boxplots of co-expression estimates against geometric mean expression levels. Gene pairs were stratified by expression levels and boxplots were used to summarize co-expression estimates in the same stratum (center line, median; box limits, upper and lower quartiles; whiskers, up to 1.5×interquartile range; points, outliers). Intervals on the x-axis denote the strata of geometric mean expression levels. $n = 1{,}828, 1{,}649, 876, 380, 159, 68, 28, 9, 2$ gene pairs in each stratum from left to right respectively for all methods except for baredSC. For baredSC, $n = 138, 303, 265, 152, 80, 37, 18, 7, 0$ gene pairs respectively. **B** Mean squared errors (MSE) against geometric mean expression levels. Genes were stratified by geometric mean expression levels and MSE were calculated for gene pairs (j, j') in the same stratum. MSE values greater than 0.25 are not shown in the figure (only found for baredSC). **C** MSE across all gene pairs in the network. Source data are provided as a Source Data file.

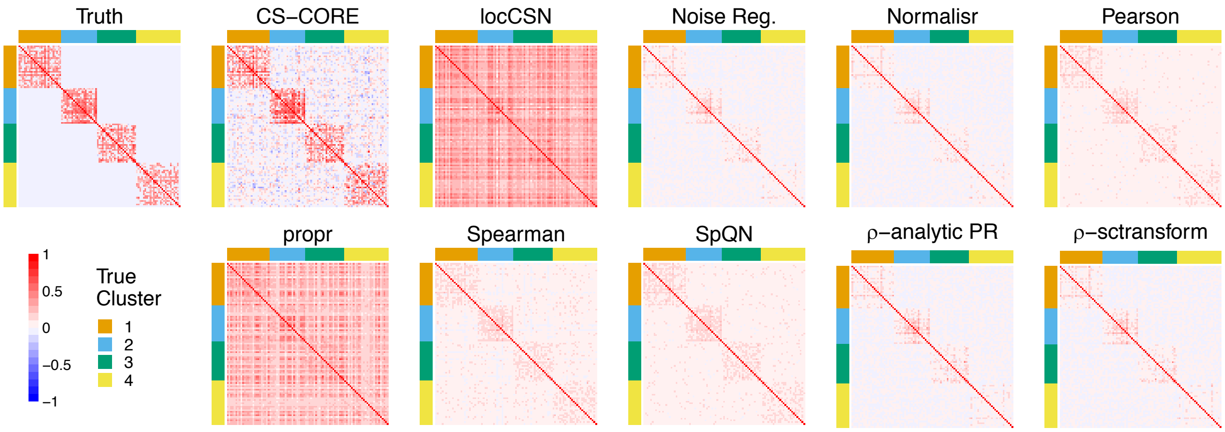

Supplementary Figure 5: Heatmaps of true and estimated co-expression networks in simulations under the same setting as Figure 4A. For all methods, genes are shown in the same order as the true co-expression network. CS-CORE is compared to locCSN, Noise Regularization, Normalisr, Pearson correlation of log normalized data (Pearson), propr, Spearman correlation of log normalized data (Spearman), SpQN, ρ-analytic PR and ρ-sctransform. Source data are provided as a Source Data file.

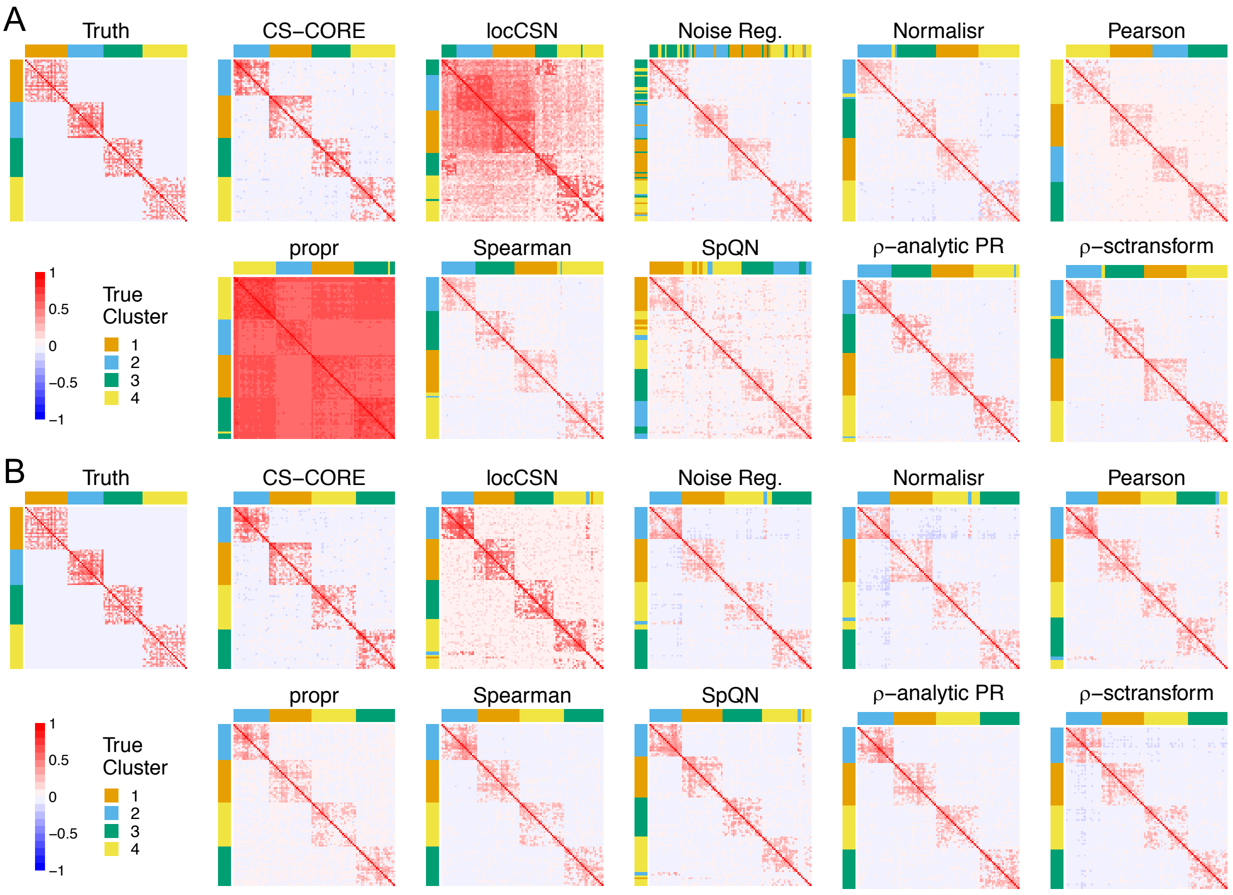

Supplementary Figure 6: Evaluation of CS-CORE in recovering co-expressed gene clusters using simulated data with **A** high gene expression levels and varying sequencing depths across cells; **B** high gene expression levels and constant sequencing depths across cells, compared to locCSN, Noise Regularization, Normalisr, Pearson correlation of log normalized data (Pearson), propr, Spearman correlation of log normalized data (Spearman), SpQN, ρ-analytic PR and ρ-sctransform. Heatmaps of true and estimated co-expression networks are shown, where genes were ordered by applying hierarchical clustering to the estimated co-expression network and color coded by their true gene cluster labels. The high expression levels were set by the top 100 highly expressed genes in excitatory neurons from [1]. The constant sequencing depth was set to 5,318. Source data are provided as a Source Data file.

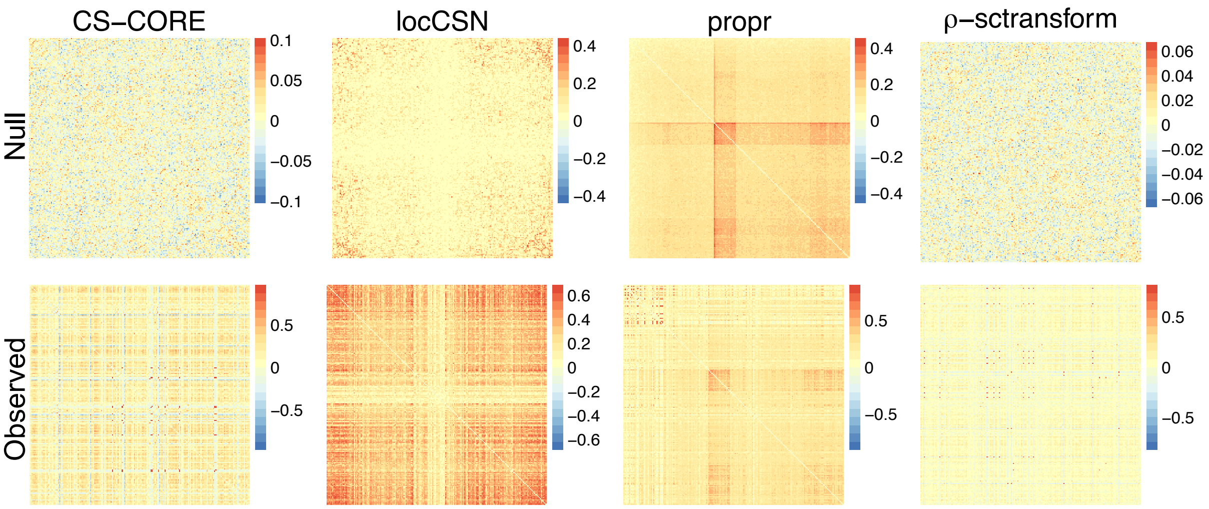

Supplementary Figure 7: Clustering structures on permuted null data and observed data for CS-CORE, locCSN, propr and ρ-sctransform. We estimated co-expression networks for the top 200 highly expressed genes using both permuted null data and observed data on excitatory neurons of control subjects from [1], where the permuted null data were generated as in Figure 1. For estimates of the same method, the genes are ordered by hierarchical clustering of the estimates based on null data. Source data are provided as a Source Data file.

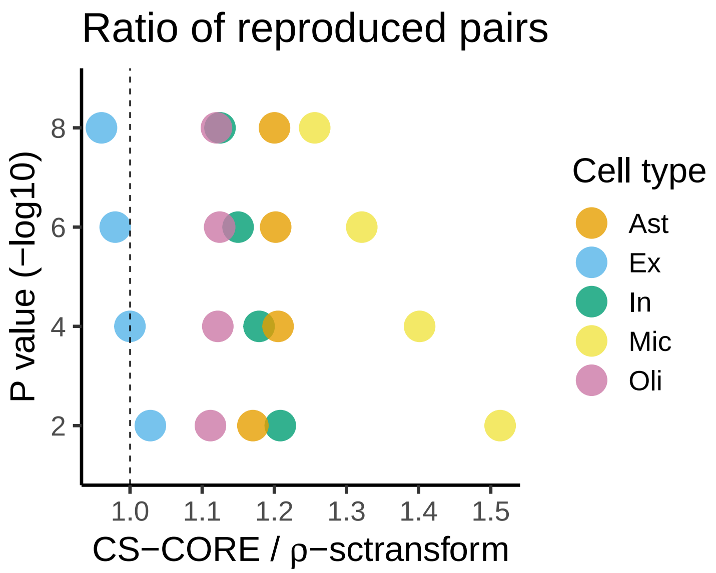

Supplementary Figure 8: Ratio of numbers of gene pairs that were identified as significant in both [1] and [15] at specified p-value cutoffs between CS-CORE and ρ-sctransform. We used the cells in five major brain cell types from control subjects from [1] and [15] respectively to estimate cell-type-specific co-expression networks. p-values were evaluated based on two-sided tests described in Methods and nominal p-values not adjusted for multiple testing were used to determine statistical significance. Source data are provided as a Source Data file.

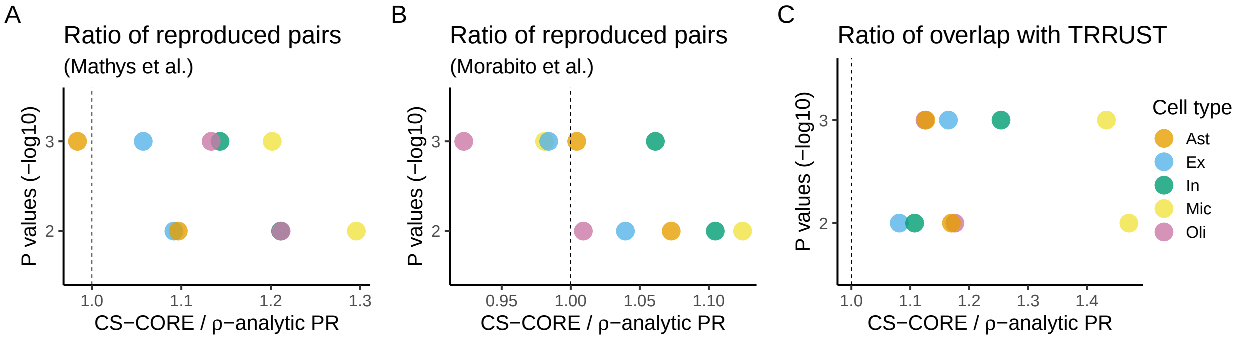

Supplementary Figure 9: Co-expression analyses using brain samples in [1]. Cell-type-specific co-expressions for five major brain cell types were estimated using CS-CORE and ρ-analytic PR (Supplementary Methods). **A** Ratio of the numbers of gene pairs that were identified as significant in both [1] and [14] at specified p-value cutoffs between CS-CORE and ρ-analytic PR. **B** Ratio of the numbers of gene pairs that were identified as significant in both [1] and [15] at specified p-value cutoffs between CS-CORE and ρ-analytic PR. **C** Ratio of the numbers of gene pairs that were identified as significant and overlapped with known TF-target gene pairs in the TRRUST database [13] between CS-CORE and ρ-analytic PR. p-values were evaluated based on two-sided tests described in Methods and nominal p-values not adjusted for multiple testing were used to determine statistical significance. Source data are provided as a Source Data file.

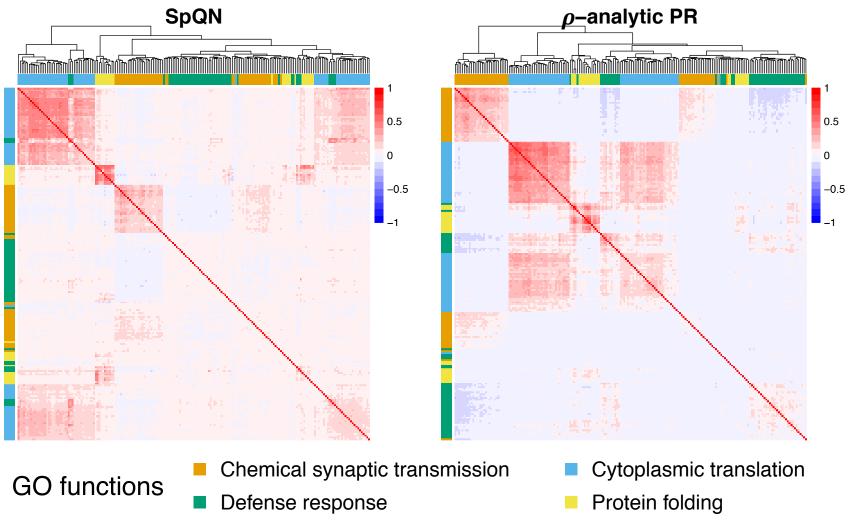

Supplementary Figure 10: Heatmaps of co-expression estimated by SpQN and ρ-analytic PR on genes from four GO terms on microglia's functions (same as Figure 5C) with genes ordered by hierarchical clustering. Source data are provided as a Source Data file.

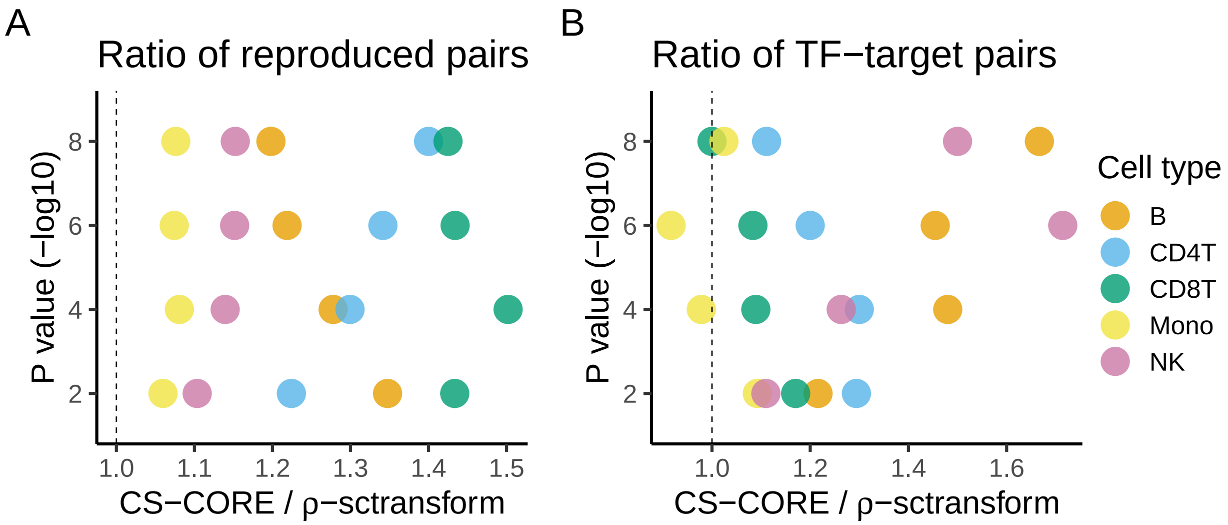

Supplementary Figure 11: Co-expression analysis using PBMC samples in [16]. We used the cells in five major immune cell types from control subjects from [16] to estimate cell-type-specific co-expression networks. **A** Ratio of the numbers of gene pairs that were identified as significant in both [16] and [17] at specified p-value cutoffs between CS-CORE and ρ-sctransform. **B** Ratio of the numbers of gene pairs that were identified as significant and overlapped with known TF-target gene pairs in the TRRUST database [13] between CS-CORE and ρ-sctransform. p-values were evaluated based on two-sided tests described in Methods and nominal p-values not adjusted for multiple testing were used to determine statistical significance. Source data are provided as a Source Data file.

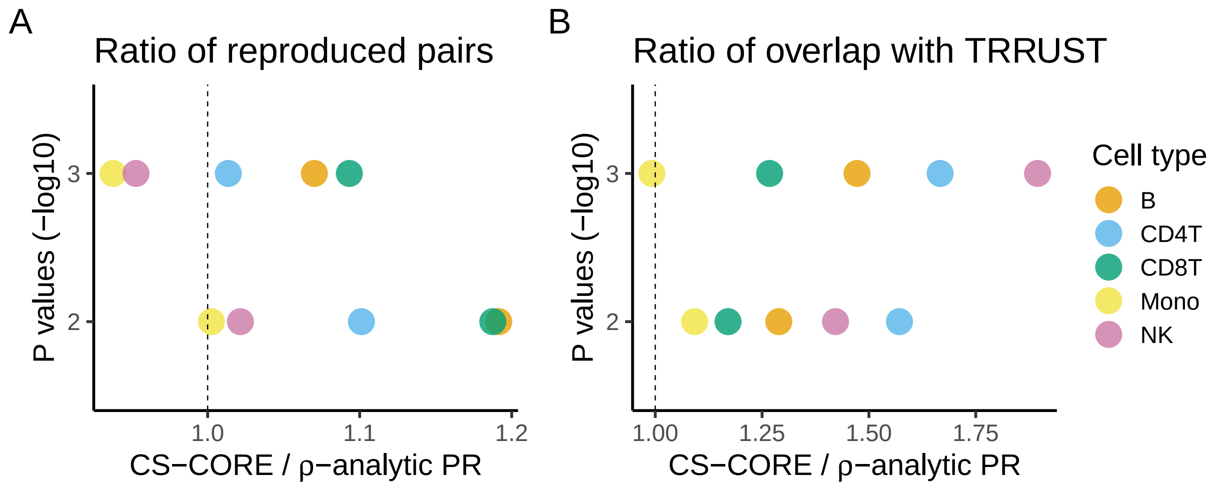

Supplementary Figure 12: Co-expression analysis using PBMC samples in [16]. We used the cells in five major immune cell types from control subjects from [16] to estimate cell-type-specific co-expression networks. **A** Ratio of the numbers of gene pairs that were identified as significant in both [16] and [17] at specified p-value cutoffs between CS-CORE and ρ-analytic PR. **B** Ratio of the numbers of gene pairs that were identified as significant and overlapped with known TF-target gene pairs in the TRRUST database [13] between CS-CORE and ρ-analytic PR. p-values were evaluated based on two-sided tests described in Methods and nominal p-values not adjusted for multiple testing were used to determine statistical significance. Source data are provided as a Source Data file.

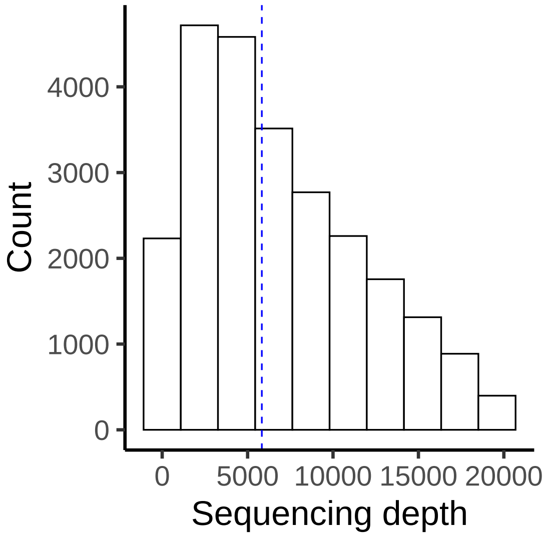

Supplementary Figure 13: Histogram of sequencing depths (sum of UMI counts in a cell) of excitatory neurons from control subjects from [1]. The median 5,833 is marked with a dashed blue line. Source data are provided as a Source Data file.

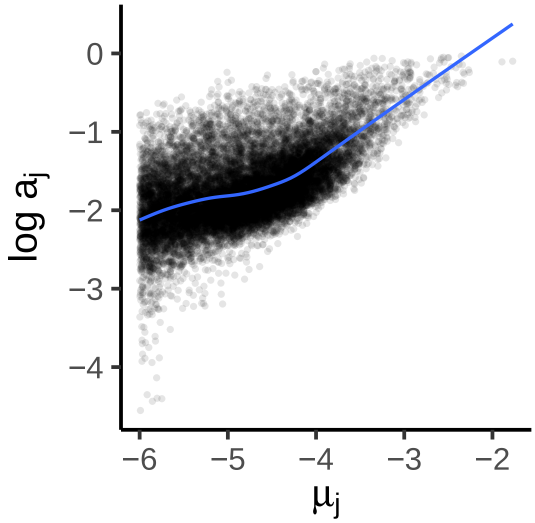

Supplementary Figure 14: Log attenuation factor $\log a_{ij}$ across expression levels μ_j in cells with sequencing depth $s_i = 2{,}000$. Marginal estimates of μ_j and CV_j were obtained as described in Supplementary Methods and a_j was calculated as $\sqrt{(s_i \mathrm{CV}_j^2)/(1/\mu_j + s_i \mathrm{CV}_j^2)}$. Source data are provided as a Source Data file.

Supplementary References

[1] Lau, S.-F., Cao, H., Fu, A. K. & Ip, N. Y. Single-nucleus transcriptome analysis reveals dysregulation of angiogenic endothelial cells and neuroprotective glia in alzheimer's disease. *Proceedings of the National Academy of Sciences* **117**, 25800–25809 (2020).

[2] Ahlmann-Eltze, C. & Huber, W. glmgampoi: fitting gamma-poisson generalized linear models on single cell count data. *Bioinformatics* **36**, 5701–5702 (2020).

[3] Hafemeister, C. & Satija, R. Normalization and variance stabilization of single-cell rna-seq data using regularized negative binomial regression. *Genome biology* **20**, 1–15 (2019).

[4] Robinson, M. D., McCarthy, D. J. & Smyth, G. K. edger: a bioconductor package for differential expression analysis of digital gene expression data. *Bioinformatics* **26**, 139–140 (2010).

[5] McCarthy, D. J., Chen, Y. & Smyth, G. K. Differential expression analysis of multifactor rna-seq experiments with respect to biological variation. *Nucleic Acids Research* **20**, 4288–97 (2012). URL `https://doi.org/10.1093/nar/gks042`.

[6] Wu, H., Wang, C. & Wu, Z. A new shrinkage estimator for dispersion improves differential expression detection in rna-seq data. *Biostatistics* **14**, 232–243 (2013). URL `https://doi.org/10.1093/biostatistics/kxs033`.

[7] Love, M. I., Huber, W. & Anders, S. Moderated estimation of fold change and dispersion for rna-seq data with deseq2. *Genome biology* **15**, 1–21 (2014).

[8] Lee, C. & Wilkinson, D. J. A review of stochastic block models and extensions for graph clustering. *Applied Network Science* **4**, 1–50 (2019).

[9] Sun, Y. & Vandenberghe, L. Decomposition methods for sparse matrix nearness problems. *SIAM Journal on Matrix Analysis and Applications* **36**, 1691–1717 (2015).

[10] Langfelder, P. & Horvath, S. Wgcna: an r package for weighted correlation network analysis. *BMC bioinformatics* **9**, 1–13 (2008).

[11] Wu, T. *et al.* clusterprofiler 4.0: A universal enrichment tool for interpreting omics data. *The Innovation* **2**, 100141 (2021).

[12] Yu, G. & He, Q.-Y. Reactomepa: an r/bioconductor package for reactome pathway analysis and visualization. *Molecular BioSystems* **12**, 477–479 (2016).

[13] Han, H. *et al.* Trrust v2: an expanded reference database of human and mouse transcriptional regulatory interactions. *Nucleic acids research* **46**, D380–D386 (2018).

[14] Mathys, H. *et al.* Single-cell transcriptomic analysis of alzheimer's disease. *Nature* **570**, 332–337 (2019).

[15] Morabito, S. *et al.* Single-nucleus chromatin accessibility and transcriptomic characterization of alzheimer's disease. *Nature Genetics* **53**, 1143–1155 (2021).

[16] Wilk, A. J. *et al.* A single-cell atlas of the peripheral immune response in patients with severe covid-19. *Nature medicine* **26**, 1070–1076 (2020).

[17] Unterman, A. *et al.* Single-cell multi-omics reveals dyssynchrony of the innate and adaptive immune system in progressive covid-19. *Nature communications* **13**, 1–23 (2022).
