# Supplementary material for: Cell-type-specific co-expression inference from single cell RNA-sequencing data
Source: Nat Commun. 2023 Aug 10;14:4846. doi: 10.1038/s41467-023-40503-7 (PMC10415381; doi:10.1038/s41467-023-40503-7)
Supplement: Supplementary file 3 — Description of Additional Supplementary Files [file 41467_2023_40503_MOESM3_ESM.pdf]

Description of Additional Supplementary Files:

Supplementary Data 1: We estimated co-expressions using CS-CORE, ρ-analytic Pearson residuals, ρ-sctransform and SpQN, respectively, extracted co-expressed gene modules and conducted GO enrichment analyses on the identified modules (see Supplementary Methods) using cells from control subjects in [1] for five major brain cell types, including astrocyte, excitatory neuron, inhibitory neuron, oligodendrocyte, and microglia. The top three GO terms with the strongest enrichment signals are presented in Supplementary Data 1 for modules with at least one highly significant GO term. One-sided hypergeometric tests were used to test the over-representation of genes from GO terms in the identified modules and GO terms with Benjamini-Hochberg adjusted p-values smaller than 0.001 were considered as significant.

Supplementary Data 2: We estimated the differential co-expression network using CS-CORE, ρ-analytic Pearson residuals, ρ-sctransform and SpQN, respectively, obtained differentially co-expressed gene modules (see Section 4.6) and conducted GO enrichment analyses on the identified modules (see Supplementary Methods) using microglia cells from control subjects and AD patients. The top three GO terms with the strongest enrichment signals are presented in Supplementary Data 2 for modules with at least one significant GO term. One-sided hypergeometric tests were used to test the over-representation of genes from GO terms in the identified modules and GO terms with Benjamini-Hochberg adjusted p-values smaller than 0.05 were considered as significant. For results from ρ-analytic Pearson residuals, ρ-sctransform and SpQN, we did not identify any GO term that captures cell-type-specific biological functions or cell-type-specific disease-related biological pathways in the presented list.

Supplementary Data 3: The analysis pipeline was the same as Supplementary Data 1 and it was performed with cells from control subjects in [2] for five major immune cell types, including B cells, CD4 positive T cells, CD8 positive T cells, monocytes and natural killer (NK) cells.

Supplementary Data 4: The analysis pipeline was the same as Supplementary Data 2 and was performed with monocytes from control subjects and COVID patients in [2].

References

[1] Lau, S.-F., Cao, H., Fu, A. K. & Ip, N. Y. Single-nucleus transcriptome analysis reveals dysregulation of angiogenic endothelial cells and neuroprotective glia in alzheimer's disease. Proceedings of the National Academy of Sciences 117, 25800–25809 (2020).

[2] Wilk, A. J. et al. A single-cell atlas of the peripheral immune response in patients with severe covid-19. Nature medicine 26, 1070–1076 (2020).
