# Supplementary material for: Cell-type-specific co-expression inference from single cell RNA-sequencing data
Source: Nat Commun. 2023 Aug 10;14:4846. doi: 10.1038/s41467-023-40503-7 (PMC10415381; doi:10.1038/s41467-023-40503-7)
Supplement: Supplementary file 5 — Reporting Summary [file 41467_2023_40503_MOESM5_ESM.pdf]

nature portfolio

Corresponding author(s): Hongyu Zhao, Jingfei Zhang

Last updated by author(s): May 29, 2023

Reporting Summary

Nature Portfolio wishes to improve the reproducibility of the work that we publish. This form provides structure for consistency and transparency in reporting. For further information on Nature Portfolio policies, see our Editorial Policies and the Editorial Policy Checklist.

Statistics

For all statistical analyses, confirm that the following items are present in the figure legend, table legend, main text, or Methods section.

n/a	Confirmed	
☐	☒	The exact sample size (*n*) for each experimental group/condition, given as a discrete number and unit of measurement
☐	☒	A statement on whether measurements were taken from distinct samples or whether the same sample was measured repeatedly
☐	☒	The statistical test(s) used AND whether they are one- or two-sided<br>*Only common tests should be described solely by name; describe more complex techniques in the Methods section.*
☒	☐	A description of all covariates tested
☐	☒	A description of any assumptions or corrections, such as tests of normality and adjustment for multiple comparisons
☐	☒	A full description of the statistical parameters including central tendency (e.g. means) or other basic estimates (e.g. regression coefficient) AND variation (e.g. standard deviation) or associated estimates of uncertainty (e.g. confidence intervals)
☐	☒	For null hypothesis testing, the test statistic (e.g. *F*, *t*, *r*) with confidence intervals, effect sizes, degrees of freedom and *P* value noted<br>*Give P values as exact values whenever suitable.*
☒	☐	For Bayesian analysis, information on the choice of priors and Markov chain Monte Carlo settings
☒	☐	For hierarchical and complex designs, identification of the appropriate level for tests and full reporting of outcomes
☐	☒	Estimates of effect sizes (e.g. Cohen's *d*, Pearson's *r*), indicating how they were calculated

Our web collection on statistics for biologists contains articles on many of the points above.

Software and code

Policy information about availability of computer code

Data collection

We evaluated the method baredSC with the implementation provided at https://baredsc.readthedocs.io/en/latest/ with default parameters.

We evaluated the method locCSN with the Python implementation provided at https://github.com/xuranw/locCSN.

We evaluated the method propR with the R package propR (v.4.2.6).

We evaluated the method rho-sctransform based on the R package Seurat (v.4.0.3).

We evaluated the Spearman (Pearson) correlation using the R package stats (v.4.1.3).

We evaluated Noise Regularization with the implementation at https://github.com/RuoyuZhang/NoiseRegularization

We evaluated Normalisr [13] with the Python implementation at https://github.com/lingfeiwang/normalisr (v.1.0.0).

We evaluated SpQN with the R package SpQN (v.1.6.0).

We performed enrichment analysis of Gene Ontology (GO) using R package clusterProfiler (v.4.2.2) and Reactome Pathway Database (Reactome) using R package ReactomePA

	(v.1.38.0).
Data analysis	The codes of the statistical method proposed in this manuscript is available on GitHub: https://github.com/ChangSuBiostats/CS-CORE, https://github.com/ChangSuBiostats/CS-CORE_python, and on Zenodo: https://doi.org/10.5281/zenodo.7983426.

For manuscripts utilizing custom algorithms or software that are central to the research but not yet described in published literature, software must be made available to editors and reviewers. We strongly encourage code deposition in a community repository (e.g. GitHub). See the Nature Portfolio guidelines for submitting code & software for further information.

Data

Policy information about availability of data

All manuscripts must include a data availability statement. This statement should provide the following information, where applicable:

- Accession codes, unique identifiers, or web links for publicly available datasets
- A description of any restrictions on data availability
- For clinical datasets or third party data, please ensure that the statement adheres to our policy

All data used in this work are publicly available. We used data from GSE157827 [https://www.ncbi.nlm.nih.gov/geo/query/acc.cgi?acc=GSE157827], syn21261143 [https://www.synapse.org/#!Synapse:syn21261143], syn22079621 [https://www.synapse.org/#!Synapse:syn22079621], COVID-19 Peripheral Blood Mononuclear Cells (PBMCs) [https://www.covid19cellatlas.org/index.patient.html], and GSE155224 [https://www.ncbi.nlm.nih.gov/geo/query/acc.cgi?acc=GSE155224]. A detailed description of the accession codes, unique identifiers or web links are included in Table 1.

For functional enrichment analysis, we used the Gene Ontology Database provided by R package clusterProfiler (v.4.2.2) and the Reactome Pathway Database provided by R package ReactomePA (v.1.38.0).

Source data are provided with this paper. Additional raw data for producing figures have been deposited in Zenodo under accession code 7983559 [https://doi.org/10.5281/zenodo.7983559].

Human research participants

Policy information about studies involving human research participants and Sex and Gender in Research.

Reporting on sex and gender	n/a
Population characteristics	n/a
Recruitment	n/a
Ethics oversight	n/a

Note that full information on the approval of the study protocol must also be provided in the manuscript.

Field-specific reporting

Please select the one below that is the best fit for your research. If you are not sure, read the appropriate sections before making your selection.

[x] Life sciences [ ] Behavioural & social sciences [ ] Ecological, evolutionary & environmental sciences

For a reference copy of the document with all sections, see nature.com/documents/nr-reporting-summary-flat.pdf

Life sciences study design

All studies must disclose on these points even when the disclosure is negative.

Sample size	No sample size calculation was performed. For simulation studies, we chose the number of cells based on the number of cells available in real data. In real data analysis, sample size was determined by existing datasets.
Data exclusions	No data were excluded from the study.
Replication	The results can be fully reproduced based on the provided source data.
Randomization	Randomization was not applicable to the study as this study was not designed to study treatment effects.
Blinding	Blinding was not applicable to the study as this study was not designed to study treatment effects.

Reporting for specific materials, systems and methods

We require information from authors about some types of materials, experimental systems and methods used in many studies. Here, indicate whether each material, system or method listed is relevant to your study. If you are not sure if a list item applies to your research, read the appropriate section before selecting a response.

Materials & experimental systems

n/a	Involved in the study
☒	☐ Antibodies
☒	☐ Eukaryotic cell lines
☒	☐ Palaeontology and archaeology
☒	☐ Animals and other organisms
☒	☐ Clinical data
☒	☐ Dual use research of concern

Methods

n/a	Involved in the study
☒	☐ ChIP-seq
☒	☐ Flow cytometry
☒	☐ MRI-based neuroimaging
